# The relationship between addiction and hikikomori tendencies: a case-control study

**DOI:** 10.3389/fpsyt.2023.1273865

**Published:** 2023-11-22

**Authors:** Peter Muris, Veerle van de Pasch, Janno van Kessel, Josine Peet

**Affiliations:** ^1^Department of Clinical Psychological Science, Faculty of Psychology and Neuroscience, Maastricht University, Maastricht, Netherlands; ^2^Changes GGZ Weert, Weert, Netherlands

**Keywords:** extreme social withdrawal, hikikomori, quality of life, addiction, social problems

## Abstract

**Aim:**

The present study examined the relationship between addiction and hikikomori (extreme social withdrawal) symptoms.

**Method:**

A group of clinically referred addiction patients (*n* = 31) and a group of age- and gender-matched non-clinical controls (*n* = 34) completed a self-report scale for measuring hikikomori symptoms (the Hikikomori Questionnaire-25) along with some other questionnaires assessing substance use (frequency and severity) and quality of life.

**Results:**

The results showed that addiction patients displayed significantly higher levels of hikikomori symptoms than the non-clinical control (Cohen's *d* = 3.41); 87.1% even showed such a high score that they were identified as being at risk for the hikikomori syndrome (vs. only 2.9% in the non-clinical control group). Correlational analyses revealed that within the addiction group, the severity of the substance use problem (as quantified by an index of craving) correlated positively with the level of hikikomori symptoms and negatively with quality of life. In other words, the more severe the addiction, the more extreme the social withdrawal tendencies and the lower the quality of life.

**Conclusion:**

Altogether, the findings provide further support for the marked social impairments of people with substance use problems and underline that this should be an important target of intervention.

## Introduction

Substance use disorders refer to a cluster of behavioral, cognitive, and physiological symptoms that are associated with the continued and heavy use of a substance despite experiencing substance-related problems that cause significant interference in daily life (1, 2). Frequently abused substances are alcohol, cannabis, stimulants, opioids, and cocaine (3), and the repeated use of all of these cause changes in the brain chemistry that are associated with the typical addiction-related phenomena of withdrawal, tolerance, dependency, craving, and relapse (4). With 12-month prevalence rates of 12% for alcohol use disorder and 2–3% for the abuse of other illicit drugs, it can be concluded that this type of problem belongs to the more common mental health problems (5). Although the aforementioned figures are based on samples of adults in the United States, there is evidence indicating that substance use disorders are also highly prevalent in other parts of the world (6).

Social problems are an important concomitant of substance use disorders. This is reflected in some of the diagnostic criteria, which refer to the detrimental consequences of the recurrent use of alcohol and drugs, such as a failure to fulfill the obligatory roles related to work or school, becoming entangled in interpersonal problems, and giving up social and recreational activities (1). For example, in a sample of 204 addicted patients who visited a drug rehabilitation center, Poudel et al. (7) found low levels of social competence in combination with high levels of difficulties in personal relationships with family members, colleagues, and peers. Moreover, it was noted that more severe substance abuse (e.g., using a substance many times per day and polydrug use) was associated with more prominent social problems. Meanwhile, it is good to keep in mind that social problems are not only a consequence of substance abuse but might also act as an antecedent facilitating the intake of alcohol and illicit drugs. In a systematic review relying on experimental and neurobiological data obtained in animal studies, Pomrenze et al. (8) summarized evidence for a bidirectional relationship. These scholars described a model in which, on the one hand, addiction promotes social difficulties and ultimately results in social isolation, and on the other hand, the stress associated with being socially isolated enhances the (continued) use of substances (9).

Hikikomori is a severe form of social withdrawal that has been originally described as a Japanese culture-bound syndrome (10), but that is also increasingly seen in other parts of the world (11). In its extreme, people with hikikomori seclude themselves most of the time in their homes for a period of minimum 6 months, are no longer engaged in education or work, and as such refrain from participation in society (12). A previous research study has indicated that hikikomori is more prevalent in persons with mental disorders including depressive disorder, social anxiety disorder, schizophrenia, and autism spectrum disorder (10, 12, 13). Furthermore, within the context of addiction, hikikomori has been predominantly linked to internet addiction, with several studies indicating that high levels of extreme social withdrawal are associated with heightened levels of problematic internet use (14–17). However, so far, only a few studies have been conducted on the relationship between substance use disorder and (extreme) social withdrawal.

In an interesting qualitative study by Tam et al. (18), 30 former drug addicts were subjected to an in-depth interview on their drug use history. Five subsequent stages of drug taking were discerned, beginning with the passive use of drugs for the social recognition of peers (stage 1) and the active use of substances to solidify relationships within the social network (stage 2), developing into a pattern of regular, habit-like abuse (stage 3), that is followed by persistent abuse of drugs characterized by social distrust and alienation (stage 4), and ultimately complete social withdrawal due to the devastating physiological and psychological damages of the prolonged drug use (stage 5). In other words, substance use problems can be perceived as a social deterioration process that starts as a prosocial phenomenon and ends as a state of “hidden drug abuse” in social isolation. In another investigation, Jeffers et al. (19) evaluated the effects of the COVID-19 pandemic on homeless people with pre-existing mental illness and substance use disorder by interviewing healthcare providers. The healthcare providers noted that the pandemic significantly increased the social isolation of their clients and that this in turn had increased the use of substances or prompted relapse in those who had been abstinent from alcohol and drugs. A final study by Chauliac et al. (20) investigated the clinical characteristics of 66 patients aged 18 to 34 years who displayed clear signs of extreme social withdrawal, which could be seen as cases of ‘hikikomori.' The results indicated that in addition to the more common psychiatric problems (i.e., affective, psychotic, and neurodevelopmental disorders), substance use problems were also quite common: 42 and 17% of these socially withdrawn individuals used cannabis and alcohol on a regular basis, respectively.

Altogether, the empirical evidence for the relation between substance use problems and (extreme) social withdrawal is still sparse. The purpose of the present study was to further examine the link between addiction problems and hikikomori symptoms. A case–control design was employed in which a group of patients who had been referred to an addiction clinic were compared with a group of age- and gender-matched non-clinical controls with regard to their scores on a self-report scale for measuring hikikomori symptoms (the Hikikomori Questionnaire-25) (21). We also administered a quality of life measure, as a previous research study has shown that both substance use problems (22, 23) and hikikomori (24, 25) are associated with lower levels of wellbeing. It was hypothesized that (1) the group of patients with addiction problems would display higher levels of hikikomori symptoms than the group of non-clinical controls, (2) the group of addiction patients would exhibit lower levels of quality of life than the non-clinical controls, and (3) there would be positive correlations between indices of addiction severity and hikikomori symptoms (only in the addiction group) and negative correlations between addiction severity and quality of life (only in the addiction group) and between hikikomori symptoms and quality of life (in both the addiction and the non-clinical control group).

## Method

### Participants and procedure

Patients of Changes GGZ in Weert, a specialized clinic for people with addiction problems, were approached by the second author (VvdP) with the request whether they would be willing to participate in a survey study on ‘addiction problems, social withdrawal, and quality of life' during the first 3 weeks of their admission. Those who were willing to participate received a link to Qualtrix, an online survey platform. The link first guided them to an information letter describing the goal and procedure of the study, which was followed by an informed consent form. After giving their consent, participants were guided to the survey, which consisted of some basic demographic questions (i.e., age and gender) and three standardized questionnaires assessing the person's substance use (problems), hikikomori symptoms, and quality of life (see below: *Assessment*). Thirty-one addiction patients were recruited in this way and completed the survey: 19 (61.3%) were men and 12 (38.7%) were women (a gender distribution which is in keeping with what has been found in previous research) (26), and their mean age was 33.54 years (*SD* = 11.61), with a range of 19–62 years.

The control participants were recruited in the social network of the second author (VvdP) by means of a snowball method. The aim was to find at least one non-addicted control participant with (about) the same age and gender for each addiction patient. The procedure was similar to that used in the addiction clinic: potential participants were approached in person and after indicating their willingness to participate, they were provided with the Qualtrix link so that they could complete the survey. Eventually, 34 non-addicted control participants, 20 men (58.8%) and 14 women (41.2%) with a mean age of 33.18 years (*SD* = 11.72, range: 19–62 years) filled in the set of questionnaires. A *t*-test and chi-square test confirmed that the addiction and the non-clinical control groups were highly comparable with regard to age [*t*(63) = 0.26, *p* = 0.80] and gender [χ^2^(1) = 0.04, *p* = 0.84].

It is important to note that none of the participants (i.e., patients and controls) who were approached for this study refused to participate or dropped out/had to be discarded (because they did not finish the survey or provided incomplete responses): this implies that the addiction patients recruited at Changes GGZ were a good representation of the population referred to this clinical facility and that there was no indication for a non-response bias in the control group (e.g., non-clinical participants with drug or social withdrawal problems refusing to participate in the study).

The study was officially approved by the Ethical Research Committee of Psychology and Neuroscience at Maastricht University, the Netherlands, as part of the research line entitled ‘The developmental psychopathology of hikikomori' that was developed by the first author (PM; reference code ERCPN-OZL_262_03_01_2023).

### Assessment

*Measurement in the Addictions for Triage and Evaluation* (MATE 2.1) (27). The MATE 2.1 is a questionnaire that can be used to assess the person's *use of psychoactive substances*, including alcohol, cannabis, opioids, cocaine, stimulants, XTC, and sedatives. For each substance, the person has to indicate the number of days of use in the past 30 days, the amount used on a typical day of use, and the total number of years of regular use. Polydrug use is also measured as the number of drugs used by a person at least once per month. Furthermore, the person also indicates the primary problem substance (i.e., the substance that is considered as causing the most problems). For the primary problem substance, five items are completed that measure the *level of craving* during the past 7 days: (1) How much of your time—when you are not using—is occupied by ideas, thoughts, impulses, or images related to using? (0 = none, 4 = more than 8 h a day); (2) How frequently do these thoughts occur? (0 = never, 4 = these thoughts are too numerous to count, and an hour rarely passes without several thoughts occurring); (3) How much distress or disturbance do these ideas, thoughts, impulses, or images related to using cause you—when you are not using? (0 = none, 4 = extreme, nearly constant, and disabling distress; (4) How much of an effort do you make to resist these thoughts or try to disregard or turn your attention away from these thoughts as they enter your mind—when you are not using? (0 = my thoughts are so minimal I don't need to actively resist them/if I do have thoughts, I always make the effort to resist them, 4 = I completely and willingly give in to all such thoughts); and (5) How strong is the drive to use [substance]? (0 = no drive to use, 4 = the drive to use is completely involuntary and overpowering). A total craving score can be obtained by summing the scores on these five items (range 0–20), with higher scores indicating higher levels of desire for the primary problem substance. Finally, MATE 2.1 measures the severity of difficulties associated with substance use in the interpersonal domain (5 items, i.e., intimate relationships, parent–child relationships, friends and family relationships, formal relationships, and other relationships) and other life areas (6 items; i.e., work/school, economic self-sufficiency, recreation activities, religious/spiritual activities, a place to live, and household tasks) by means of a 4-point Likert scale with 0 = none/not applicable and 4 = very severe. The MATE 2.1 has been demonstrated to be a reliable and valid scale for measuring addictive behavior, craving, and associated problems (28, 29). In this study, the Cronbach's alphas of the MATE 2.1 craving score and the associated problem scales ranged between 0.63 to 0.89 for addiction patients and between 0.37 (problems in other life areas) and 0.78 (craving) for non-clinical control participants.

The *25-item Hikikomori Questionnaire* (HQ-25) (21) has been developed to measure the intensity of social withdrawal symptoms during the past 6 months. Items have to be rated on a 5-point Likert scale with 0 = strongly disagree and 4 = strongly agree. In addition to a total score (range 0–100), three factors have been consistently identified, namely, socialization problems (e.g., “I stay away from other people” or “I feel uncomfortable around other people”), isolation (e.g., “I shut myself in my room” or “I have little contact with other people talking, writing, and so on”), and lack of emotional support [e.g., “There really is not anyone with whom I can discuss matters of importance” or “I can share my personal thoughts with several people” (reversed item)]. The reliability of the HQ-25 is excellent, and this was also true in the current study: Cronbach's alphas for the total score were 0.96 in addiction patients and 0.89 in non-clinical control participants, and internal consistency coefficients ranged between 0.67 and 0.94 for the three subscales. Furthermore, there is evidence for the validity of the measure; that is, the scale was found to correlate in the predicted way with measures of loneliness, social support, and mental health problems, and differentiated well between people with a hikikomori vs. a “normal” lifestyle (21, 30, 31).

The short version of the *Mental Health Quality of Life* questionnaire (MHQoL) (32) is a 7-item self-report scale measuring people's quality of life in the domains of self-image, independence, mood, relationships, daily activities, physical health, and future. Each item has four response levels, with 0 indicating “very dissatisfied” and 3 indicating “very satisfied.” Thus, MHQoL total scores vary from 0 to 21, with higher scores reflecting a better quality of life. One additional item assesses the self-rated overall psychological wellbeing using an 11-point scale ranging from 0 (“worst imaginable psychological wellbeing”) to 10 (“best imaginable psychological wellbeing”). The psychometric properties have been investigated in a mixed sample of 479 mental health patients and 110 members of the general population (33). Results indicated that the scale has excellent internal consistency (Cronbach's α = 0.85) and test–retest reliability (*r* = 0.85), and good convergent (i.e., substantial positive correlations with alternative quality of life measures), divergent (i.e., substantial negative correlation with psychopathology index), and discriminant validity (i.e., satisfactory discrimination between patients and non-patients). In the present study, the total MHQoL score had a Cronbach's alpha of 0.90 in addiction patients and 0.82 in non-clinical control participants.

### Statistical analyses

The *Statistical Package for the Social Scienc*es was used to obtain descriptive statistics and Cronbach's alphas of various questionnaires. To compare the addiction and non-clinical control groups on various dependent variables, Mann–Whitney *U*-tests (for the non-normally distributed MATE 2.1 “use of psychoactive substances” data), independent sample *t*-tests (for the other more continuous questionnaire scores), and crosstabs chi-square tests (in case of categorical data) were used. Furthermore, correlations were computed within the groups of addiction patients and non-clinical control participants to study relations among addiction severity indices, hikikomori symptoms, and quality of life scores.

## Results

### Clinical characteristics of addiction patients

Table 1 displays an overview of the primary problem substance as reported on the MATE 2.1 by the 31 addiction patients. As can be seen, alcohol (*n* = 8, 25.8%) and cocaine (*n* = 7, 22.6%) were reported as the main substances of abuse, followed by cannabis, ketamine, and speed (*n*'s between 3 and 5, 9.7–16.1%) and, finally, tranquilizer, methadone, and GHB, which were less frequently used (*n* = 1, 3.2%).

**Table 1:** Primary problem substance (i.e., the substance that is considered as causing the most problems) as indicated on the MATE 2.1 by the addiction patients (*N* = 31).

**Substance**	***n***	**%**
Alcohol	8	25.8
Cocaine	7	22.6
Cannabis	5	16.1
Ketamine	5	16.1
Speed	3	9.7
Tranquilizer	1	3.2
Methadone	1	3.2
Gamma-hydroxybutyrate (GHB)	1	3.2
Polydrug use^†^	28	90.3

MATE 2.1, Measurement in the Addictions for Triage and Evaluation, version 2.1. ^†^Number of substances used at least once per month.

As predicted, the addiction patients generally indicated that they had used various types of substances more frequently during the past month, used greater amounts of the substances on a typical day of use, and had used these substances for a greater number of years as compared to the non-clinical control participants, although—due to the low frequency of use of some substances—not all differences were statistically significant (see upper panel of Table 2). Most importantly, significant between-group differences were noted on the MATE 2.1 severity indexes; that is, addiction patients displayed significantly higher levels of craving for the substance [*t*(47.42) = 17.57, *p* < 0.001, Cohen's *d* = 4.42] as well as higher levels of substance use-associated social [*t*(33.95) = 16.93, *p* < 0.001, *d* = 4.29] and non-social problems [*t*(35.17) = 13.86, *p* < 0.001, *d* = 3.50] as compared to non-clinical controls.

**Table 2:** Descriptive statistics (means and standard deviations) of various measures in addiction patients and non-clinical controls.

**MATE 2.1 use of psychoactive substances**	**Addiction patients (*n* = 31)**	**Non-clinical controls (*n* = 34)**	**Mann-Whitney Test (*U*)**	***p***
Alcohol, number of days used per 30 days	10.45 (9.82)	5.74 (5.64)	397.5	0.09
Alcohol, amount on a day of use	7.74 (7.00)	3.68 (3.11)	362	<0.05
Alcohol, years of regular use	2.23 (6.37)	2.32 (4.00)	500	0.67
Cannabis, number of days used per 30 days	9.16 (12.53)	0.35 (1.10)	334	<0.01
Cannabis, amount on a day of use	0.87 (1.18)	0.06 (0.24)	343	0.001
Cannabis, years of regular use	1.97 (4.42)	0.00 (0.00)	391	<0.01
Opiates, number of days used per 30 days	1.03 (5.38)	0.00 (0.00)	476	0.07
Opiates, amount on a day of use	0.77 (3.61)	0.00 (0.00)	476	0.07
Opiates, years of regular use	0.10 (0.54)	0.00 (0.00)	510	0.3
Cocaine, number of days used per 30 days	5.71 (8.49)	0.15 (0.56)	334	0.001
Cocaine, amount on a day of use	0.71 (1.10)	0.03 (0.17)	351.5	0.001
Cocaine, years of regular use	1.06 (3.13)	0.00 (0.00)	425	<0.01
Stimulants, number of days used per 30 days	5.23 (7.65)	0.03 (0.17)	298	<0.001
Stimulants, amount on a day of use	0.94 (1.12)	0.00 (0.00)	289	<0.001
Stimulants, years of regular use	0.39 (1.52)	0.00 (0.00)	476	0.07
MDMA/Ecstasy, number of days used per 30 days	0.90 (2.32)	0.15 (0.36)	490	0.46
MDMA/Ecstasy, amount on a day of use	0.42 (1.06)	0.12 (0.33)	479	0.32
MDMA/Ecstasy, years of regular use	0.39 (2.16)	0.00 (0.00)	510	0.3
Sedatives, number of days used per 30 days	4.39 (9.16)	0.71 (2.88)	368.5	<0.01
Sedatives, amount on a day of use	1.35 (3.68)	0.06 (0.24)	364	<0.01
Sedatives, years of regular use	0.32 (1.14)	0.12 (0.54)	505.5	0.54
Other substances, number of days used per 30 days	2.61 (7.07)	0.00 (0.00)	408	<0.01
Other substances, amount on a day of use	1.03 (4.48)	0.00 (0.00)	425	<0.01
Other substances, years of regular use	0.45 (1.29)	0.00 (0.00)	442	<0.05
Polydrug use (# used at least once per month)	2.94 (1.31)	1.32 (0.91)	151.5	<0.001
**MATE 2.1 Severity of problem**			**Independent samples test (** ***t*** **)**	***p***
Level of craving for substance^†^	15.19 (3.77)	1.56 (2.20)	17.57	<0.001
Substance use-related social problems	14.10 (4.28)	0.65 (1.15)	16.93	<0.001
Substance use-related other problems	13.36 (4.72)	1.12 (1.45)	13.86	<0.001
**MHQoL**				
Quality of life	4.32 (4.27)	16.59 (3.14)	13.27	<0.001
Overall psychological wellbeing	2.52 (1.57)	7.56 (1.37)	13.81	<0.001
**HQ-25**				
Total score	71.26 (20.80)	15.38 (10.15)	13.56	<0.001
Socialization problems	32.52 (9.83)	7.21 (5.49)	12.64	<0.001
Isolation	22.61 (7.43)	4.18 (3.25)	12.75	<0.001
Lack of emotional support	16.13 (5.00)	4.00 (3.42)	11.3	<0.001

MATE 2.1, Measurement in the Addictions for Triage and Evaluation, version 2.1. MDMA, Methylenedioxy-methamphetamine; MHQoL, Mental Health Quality of Life questionnaire. HQ-25 = 25-item Hikikomori Questionnaire. ^†^In addiction patients, craving scores were provided for the primary problem substance; in non-clinical controls, craving scores were obtained for the substance most frequently used.

As an indicator of the severity of the addiction, it was found that 26 patients (83.9%) displayed a craving score of 12 or higher on the MATE 2.1 (27), which is indicative of clinically problematic substance use [none of the participants, i.e., 0.0% in the control group met this criterion; χ^2^(1) = 47.53, *p* < 0.001]. In a similar vein, polydrug use was clearly more present in the addiction patients: 90.3% of them used more than one drug at least once per month, whereas this percentage was only 29.4% in the control group [χ^2^(1) = 24.77, *p* < 0.001].

### Hikikomori symptoms in addiction patients vs. non-clinical controls

**Figure 1:**
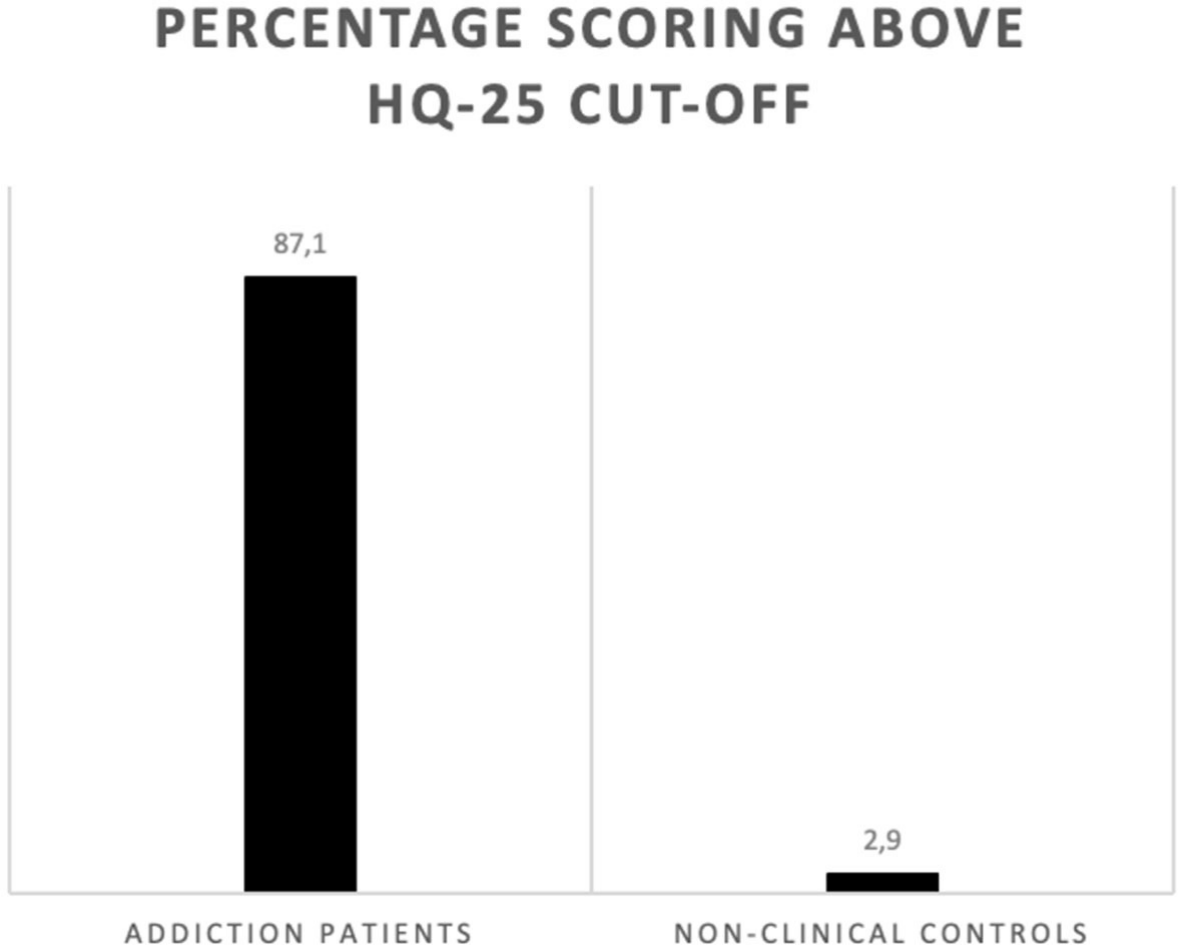
Percentages of participants in both groups reporting severe hikikomori symptoms. *n* = 31 for addiction patients and *n* = 34 for non-clinical controls. HQ-25 = 25-item Hikikomori Questionnaire. The used cutoff score was 42 (21).

An independent sample *t*-test was conducted to compare the HQ-25 scores of the addiction patients and non-clinical controls. The results indicated that the addiction patients displayed statistically significantly higher levels of hikikomori symptoms than the control participants, and this difference was not only found on the HQ-25 total score [*t*(42.62) = 13.56, *p* < 0.001, *d* = 3.41] but also on the three subscales of socialization problems [*t*(46.10) = 12.64, *p* < 0.001, *d* = 3.18], isolation [*t*(40.28) = 12.75, *p* < 0.001, *d* = 3.21], and lack of social support [*t*(52.36) = 11.30, *p* < 0.001, *d* = 2.83] (see lower panel of Table 2). Using Teo et al.'s (21) cutoff score of 42 to identify individuals at risk for hikikomori syndrome, it was found that 27 addiction patients (87.1%) showed this type of extreme social withdrawal vs. only one participant in the non-clinical control group (2.9%) [χ^2^(1) = 46.83, *p* < 0.001] (see Figure 1).

Additional analyses were conducted within the addiction group to explore differences in hikikomori symptom scores between patients who indicated abuse of a legal substance (i.e., alcohol and cannabis; *n* = 13) and patients who indicated abuse of an illegal substance (i.e., cocaine, ketamine, speed, tranquilizer, methadone, and GHB; *n* = 18) as their main problem. Independent samples *t*-tests did not reveal any significant differences between the legal and illegal abuse groups [e.g., HQ-25 total scores being 73.62, *SD* = 18.25 vs. 69.56, *SD* = 22.83, respectively; *t*(29) = 0.53, *p* = 0.60].

### Quality of life in addiction patients vs. non-clinical controls

A comparison of the MHQoL scores of both groups showed that addiction patients reported statistically significant lower levels of quality of life and overall psychological wellbeing as compared to the non-clinical control participants [*t*(63) = 13.27, *p* < 0.001, *d* = 3.27 and *t*(63) = 13.81, *p* < 0.001, *d* = 3.42, respectively] (see lower panel of Table 2).

### Correlations among addiction severity, hikikomori symptoms, and quality of life

Table 3 shows the correlations between MATE 2.1 addiction severity indices, MHQoL, and HQ-25 scores as computed for addiction patients and non-clinical controls separately. As can be seen, in the group of addiction patients, statistically significant positive correlations were found between MATE 2.1 craving and substance-related social and non-social problems scales on the one hand and various hikikomori symptoms scores were noted (*r*'s between 0.51 and 0.67, *p*'s <0.01), whereas significant negative correlations were documented between addiction severity and quality of life (*r*'s between −0.47 and −0.69, *p*'s <0.01) and between hikikomori symptoms and quality of life scores (*r*'s between −0.58 and −0.87, *p*'s <0.01). Polydrug use, which could be seen as another indicator of addiction severity, was significantly correlated with MATE 2.1 social problems *(r* = −0.48, *p* < 0.01). However, correlations between polydrug use and hikikomori symptoms and quality of life scores did not attain statistical significance.

**Table 3:** Correlations between scores on various questionnaires computed separately for addition patients (below the diagonal) and non-clinical control (above the diagonal).

	**(1)**	**(2)**	**(3)**	**(4)**	**(5)**	**(6)**	**(7)**	**(8)**	**(9)**
**MATE 2.1 severity of problem**									
(1) Level of craving for substance^†^		0.27	0.24	−0.10	−0.02	0.39^*^	0.32	0.37^*^	0.29
(2) Substance use-related social problems	0.63^***^		0.48^**^	−0.27	−0.22	0.28	0.20	0.11	0.42^*^
(3) Substance use-related other problems	0.61^***^	0.69^***^		−0.20	−0.05	0.30	0.25	0.30	0.22
**MHQoL**									
(4) Quality of life	−0.69^***^	−0.47^**^	−0.55^**^		0.76^***^	−0.51^**^	−0.44^*^	−0.45^**^	−0.38^*^
(5) Overall psychological wellbeing	−0.53^**^	−0.53^**^	−0.64^***^	0.80^***^		−0.36^*^	−0.35^*^	−0.24	−0.28
**HQ-25**									
(6) Total score	0.65^***^	0.58^**^	0.62^***^	−0.84^***^	−0.79^***^		0.94^***^	0.80^***^	0.70^***^
(7) Socialization problems	0.67^***^	0.56^**^	0.59^***^	−0.87^***^	−0.78^***^	0.97^***^		0.71^***^	0.50^***^
(8) Isolation	0.60^***^	0.52^**^	0.56^**^	−0.79^***^	−0.72^***^	0.97^***^	0.96^***^		0.30
(9) Lack of emotional support	0.51^**^	0.56^**^	0.58^**^	−0.58^**^	−0.68^***^	0.80^***^	0.66^***^	0.68^***^	

n = 31 for addiction patients, n = 34 for clinical controls. MATE 2.1, Measurement in the Addictions for Triage and Evaluation, version 2.1; MHQoL, Mental Health Quality of Life questionnaire. HQ-25 = 25-item Hikikomori Questionnaire. ^***^ p <0.001, ^**^ p <0.01, ^*^ p <0.05.

To explore whether the social problems of the addiction patients reflected ‘true' hikikomori symptoms or were mainly due to the abuse of drugs, a partial correlation was computed between addiction severity as measured by the MATE 2.1 craving score and the HQ-25 total score, while controlling for MATE 2.1 social problems. A statistically significant positive (partial) correlation of 0.45 (*p* < 0.01) was found, which suggests that the extreme social withdrawal tendencies as reported by the addiction patients were not merely reflecting social difficulties caused by the use of addictive substances.

In the control participants, addiction severity indices were less clearly related to hikikomori symptoms and quality of life scores probably due to predominantly low scores and low variability on the MATE 2.1 in this non-clinical group. Nevertheless, the few significant correlations that were found appeared to be in the predicted direction; that is (non-clinical), craving was positively correlated with the HQ-25 total score (*r* = 0.39, *p* < 0.05), and in particular, the subscale ‘isolation' (*r* = 0.37, *p* < 0.05), while substance use-related social problems were positively associated with the subscale ‘lack of social support' (*r* = 0.42, *p* < 0.05). Furthermore, even in these non-clinical participants, the level of hikikomori symptoms was negatively associated with quality of life scores, especially with the MHQoL total score (*r*'s between −0.38 and −0.51, *p*'s <0.05).

## Discussion

The present study examined the relationship between addiction and hikikomori by using a case–control design: a survey was administered in a group of patients who had been referred to an addiction clinic and a group of age- and gender-matched non-clinical control participants. The patients abused a variety of substances, of which alcohol, cocaine, cannabis, ketamine, and speed were the most common. Scores on the MATE 2.1, a standardized measure of addiction, generally indicated that the addiction patients displayed higher frequencies of (poly)substance use than the non-clinical control participants and also that this use was associated with higher levels of craving and associated social and non-social problems. In absolute terms, the vast majority of the addiction patients (i.e., 83.9%) reported craving scores in the clinical range (27), which was not that surprising given that they were assessed during the first 3 weeks in the clinic when they still experienced significant physiological and/or emotional withdrawal symptoms. A comparison of the characteristics of the addiction patients included in this study with figures provided by the National Alcohol and Drugs Information System (describing 54,865 clients who were seeking help in 2021 for an addiction problem) (34) revealed that they were a good representation of the addiction population in the Netherlands.

Clear support was found for the hypothesis that the group of patients with addiction problems would display higher levels of hikikomori symptoms than the group of non-clinical controls. More specifically, addiction patients exhibited substantially higher scores on the total score of the HQ-25 as well as on all three of its subscales referring to socialization problems, isolation, and lack of emotional support than the non-clinical control participants. Furthermore, it was found that 87.1% of the addiction patients had a score of 42 or higher on the HQ-25, which is indicative of being at risk for prolonged and extreme social withdrawal (21). Because this was a correlational study, we cannot draw conclusions about the directionality of the observed relation between addiction and hikikomori. On the one hand, it may well be that addiction problems fuel withdrawal tendencies, which ultimately result in (extreme) social isolation (18) and feelings of loneliness (35), but on the other hand, it is also possible that the use of substances reflects a way of coping with the negative feelings associated with the lack of contact with other people (36). Meanwhile, it is good to keep in mind that there may be truth in both scenarios as some scholars have put forward that addicted individuals might be trapped in a vicious circle in which substance use, social withdrawal, and isolation mutually strengthen each other (8, 9).

One could argue that the use of some substances is difficult to reconcile with a picture of extreme social withdrawal as the total confinement to one's home would hinder the person from visiting locations where he/she can acquire the needed illicit drugs. A comparison of the hikikomori scores between patients who were mainly addicted to legal substances (alcohol and cannabis) and patients who predominantly abused illegal substances (various types of “hard drugs”) did not support this line of reasoning as no significant differences in HQ-25 scores were noted. Meanwhile, it should be borne in mind that it is currently quite easy to acquire alcohol and drugs, even without having to leave the house. In recent years, in particular, since the COVID-19 pandemic, we have seen the rise of online services, which makes it possible to order the delivery of all kinds of legal and illegal substances at home (37).

The addiction patients clearly displayed lower levels of quality of life than the non-clinical control participants. The patients reported an average score of 4.32 on the MHQoL, which is clearly lower than the cut-off score of 12 which is considered indicative of a poor quality of life (33). This was confirmed by the overall self-rating of their psychological wellbeing: the mean score of only 2.52 out of 10 demonstrated that the addicted patients indicated to experience very little positive emotions such as happiness and perceived their life as rather meaningless and purposeless. Altogether, these findings are in keeping with previous studies on the low quality of life of individuals with substance use problems (22, 23). Especially at the beginning of their clinical admission, addiction patients often experience a negative state that is characterized by abstinence-related physical symptoms and associated feelings of depression and hopelessness (38).

The correlational analysis that was conducted in both groups separately generally yielded the expected results. In the addiction group, the severity of the substance use problem—as quantified by the MATE 2.1 craving index—correlated positively with the level of hikikomori symptoms and negatively with quality of life. In other words, the more severe the addiction, the more extreme the social withdrawal tendencies and the lower the quality of life. Interestingly, the result of an additional analysis (in which we computed a correlation between craving and hikikomori scores while controlling for addiction-related social problems) indicated that the high extreme social withdrawal levels displayed by the addiction patients could not be fully attributed to the social problems arising from the substance abuse. This provides an indication that at least part of addiction patients truly display signs of the hikikomori syndrome. Notably, correlations between polydrug use and hikikomori symptoms/quality of life were non-significant. This was probably due to the rather liberal definition of polydrug use (i.e., use of multiple substances at least once per month), which made it a less valid index of addiction severity. In the non-clinical control group, correlations between substance use indicators and hikikomori symptoms/quality of life were either non-significant or considerably smaller than those noted in the addiction group, which is probably due to the fact that craving ratings were rather low in these non-addicted participants, implying that there was little variation in scores which is needed to find more substantial correlations.

In both addiction and the non-clinical control groups, significant negative correlations were noted between hikikomori symptoms and quality of life. Thus, the more the participants reported a tendency toward social withdrawal and isolation, the poorer they evaluated their quality of life and psychological health. This finding aligns well with the notion that human beings generally want to form and maintain stable interpersonal relationships (39) and that any frustration of this “need to belong” (whether “big” in the case of the addiction patients or “smaller” in the case of the non-clinical control participants) undermines people's general sense of wellbeing (40).

Apart from the cross-sectional design and the accompanying fact that no cause-effect conclusions can be drawn regarding the relation between addiction and hikikomori symptoms, the present study suffered from various other limitations. First, the clinical sample was relatively small and consisted of patients who were abusing a variety of substances and who were seeking treatment for their problems, all of which may have implications for the generalizability of the results. For example, the use of some substances might be associated with more severe social withdrawal effects than the use of other substances (41). Furthermore, social problems are an important determinant of the help-seeking behavior of addicted patients (42), and this might explain why so many of them exhibited clinically elevated hikikomori symptoms. Second, the non-clinical control group was matched on gender and age with the addiction group, but it remains unclear whether both groups were comparable with regard to other demographic (e.g., socioeconomic status) or clinical (e.g., social anxiety or attention-deficit/hyperactivity problems) characteristics that have been found to be significantly related to substance use problems (43–45). Third, the study solely relied on participants' self-report. There is evidence that self-reports on the frequency and severity of substance use are often biased (46) and that this is also true for addiction patients' reports of characteristics of their social network (47). Fourth, the study was conducted in the Netherlands, which is a country with a rather lenient policy regarding alcohol and drugs (e.g., the sale of cannabis is permitted and the possession of small amounts of drugs for personal use is tolerated). Obviously, many other countries have more stringent rules, and it seems important to study the effect of more strict drug policies on the relationship between addiction and hikikomori.

In conclusion, the present study found a clear relation between addiction and the tendency to extreme social withdrawal also known as hikikomori. Future longitudinal investigations should make an attempt to unravel the dynamic relationship between substance use problems and social withdrawal and loneliness. In the meantime, given the low quality of life levels associated with both addiction and hikikomori, interventions for substance use disorder should not only focus on alcohol and drug rehabilitation and the promotion of psychological resilience in addicted persons but also include components that aim to strengthen their social networks and help them out of their social isolation (48, 49).

## Data availability statement

The raw data supporting the conclusions of this article will be made available by the authors, without undue reservation.

## Ethics statement

The studies involving humans were approved by the Ethical Research Committee of Psychology and Neuroscience, Maastricht University, Netherlands. The studies were conducted in accordance with the local legislation and institutional requirements. The participants provided their written informed consent to participate in this study.

## Author contributions

PM: Conceptualization, Formal analysis, Investigation, Methodology, Writing—original draft. VP: Conceptualization, Formal analysis, Investigation, Writing—review & editing. JK: Conceptualization, Investigation, Writing—review & editing. JP: Conceptualization, Investigation, Writing—review & editing.

## References

[1] American Psychiatric Association. Diagnostic and Statistical Manual of Mental Disorders, 5th Edn. Washington, DC: American Psychiatric Association (2022).

[2] Rehm J Marmet S Anderson P. Defining substance use disorders: Do we need really more than heavy use? Alcohol Alcohol. (2013) 48:633–40. 10.1093/alcalc/agt127 23926213

[3] Ignaszewski MJ. The epidemiology of drug abuse. J Clin Pharmacol. (2021) 61:S10–7. 10.1002/jcph.1937 34396554

[4] Volkow ND Koob GF McLellan AT. Neurobiologic advances from the brain disease model of addiction. N Engl J Med. (2016) 374:363–71. 10.1056/NEJMra1511480 26816013 PMC6135257

[5] Merikangas KR McClair VL. Epidemiology of substance use disorders. Hum Genet. (2012) 131:779–89. 10.1007/s00439-012-1168-0 22543841 PMC4408274

[6] Castaldelli-Maia JM Bhugra D. Analysis of global prevalence of mental and substance use disorders within countries: focus on sociodemographic characteristics and income levels. Int Rev Psychiatry. (2022) 34:6–15. 10.1080/09540261.2022.2040450 35584016

[7] Poudel A Sharma C Gautam S Poudel A. Psychosocial problems among individuals with substance use disorders in drug rehabilitation centers, Nepal. Subst Abuse: Treat Prev Policy. (2016) 11:28. 10.1186/s13011-016-0072-3 27528233 PMC4986270

[8] Pomrenze MB Paliarin F Maiya R. Friend of the devil: negative social influences driving substance use disorders. Front Behav Neurosci. (2022) 16:836996. 10.3389/fnbeh.2022.836996 35221948 PMC8866771

[9] Christie NC. The role of social isolation in opioid addiction. Soc Cogn Affect Neurosci. (2021) 16:645–56. 10.1093/scan/nsab029 33681992 PMC8259283

[10] Teo AR. A new form of social withdrawal in Japan: a review of hikikomori. Int J Soc Psychiatry. (2010) 56:178–85. 10.1177/0020764008100629 19567455 PMC4886853

[11] Kato TA Tateno M Shinfuku N. Does the ‘hikokomori' syndrome of social withdrawal exist outside Japan? A preliminary international investigation. Soc Psychiatry Psychiatr Epidemiol. (2012) 47:1061–75. 10.1007/s00127-011-0411-7 21706238 PMC4909153

[12] Teo AR Gaw AC. Hikikomori, a Japanese culture-bound syndrome of social withdrawal? A proposal for DSM-5. J Nerv Ment Dis. (2010) 198:444–9. 10.1097/NMD.0b013e3181e086b1 20531124 PMC4912003

[13] Muris P Ollendick TH. Contemporary hermits: a developmental psychopathology account of extreme social withdrawal (hikikomori) in young people. Clin Child Fam Psychol Rev. (2023) 26:459–81. 10.1007/s10567-023-00425-8 36653555 PMC9848719

[14] Amendola S Cerutti R Presaghi F. Hikikomori, problematic internet use and psychopathology: correlates in non-clinical and clinical samples of young adults in Italy. J Psychopathol. (2021) 27:106–14. 10.36148/2284-0249-412

[15] Kato TA Shinfuku N Tateno M. Internet society, internet addiction, and pathological social withdrawal: the chicken and egg dilemma for internet addiction and hikikomori. Curr Opin Psychiatry. (2020) 33:264–70. 10.1097/YCO.0000000000000601 32101902

[16] Stip E Thibault A Beauchamp-Chatel A Kisely S. Internet addiction, hikikomori syndrome, and the prodromal phase of psychosis. Front Psychiatry. (2016) 7:6. 10.3389/fpsyt.2016.00006 26973544 PMC4776119

[17] Tateno M Teo AR Ukai W. Internet addiction, smartphone addiction, and hikikomori trait in Japanese young adult: social isolation and social network. Front Psychol. (2019) 10:455. 10.3389/fpsyt.2019.00455 31354537 PMC6635695

[18] Tam CH Kwok SI Lo TW Lam SH Lee GK. Hidden drug use in Hong Kong: from social acquaintance to social isolation. Front Psychiatry. (2018) 9:457. 10.3389/fpsyt.2018.00457 30319464 PMC6167475

[19] Jeffers A Meehan AA Barker J. Impact of social isolation during the COVID-19 pandemic on mental health, substance use, and homelessness: qualitative interviews with behavioral health providers. Int J Environ Res Public Health. (2022) 19:12120. 10.3390/ijerph191912120 36231422 PMC9566547

[20] Chauliac N Couillet A Faivre S Brochard N Terra JL. Characteristics of socially withdrawn youth in France: a retrospective study. Int J Soc Psychiatry. (2017) 63:339–44. 10.1177/0020764017704474 28446040

[21] Teo AR Chen JI Kubo H. Development and validation of the 25-bibitem hikokomori questionnaire (HQ-25). Psychiatry Clin Neurosci. (2018) 72:780–8. 10.1111/pcn.12691 29926525 PMC6221010

[22] Armoon B Fleury MJ Bayat AH Bayani A Mohammadi R Griffiths MD. Quality of life and its correlated factors among patients with substance use disorders: a systematic review and meta-analysis. Arch Public Health. (2022) 80:179. 10.1186/s13690-022-00940-0 35927697 PMC9351239

[23] Rudolf H Watts J. Quality of life in substance abuse and dependency. Int Rev Psychiatry. (2002) 14:190–7. 10.1080/09540260220144975

[24] Nonaka S Sakai M. The effect of hikikomori on quality of life. Jpn J Psychol. (2014) 85:313–8. 10.4992/jjpsy.85.13315 25272449

[25] Nonaka S Sakai M. Measuring the quality of life for individuals with prolonged social withdrawal (hikikomori). Psychiatry Investig. (2022) 19:341–7. 10.30773/pi.2021.0348 35505459 PMC9136523

[26] McHugh RK Votaw VR Sugarman DE Greenfield SF. Sex and gender differences in substance use disorders. Clin Psychol Rev. (2018) 66:12–23. 10.1016/j.cpr.2017.10.012 29174306 PMC5945349

[27] Schippers GM Broekman TG Buchholz A. MATE 2.1. Manual and Protocol. Nijmegen: Bêta Boeken (2011).

[28] Oudejans S De Weert-Van Oene G Spits M. A self-reported version of the measurements in the addictions for triage and evaluation-Q: concurrent validity with the MATE 21. Eur Addict Res. (2020) 26:20–7. 10.1159/000503625 31639811 PMC6979419

[29] Schippers GM Broekman TG Buchholz A Koeter MWJ Van den Brink W. Measurements in the addictions for triage and evaluation (MATE): an instrument based on the WHO family of international classifications. Addict. (2010) 105:862–71. 10.1111/j.1360-0443.2009.02889.x 20331557

[30] Amendola S Presaghi F Teo AR Cerutti R. Psychometric properties of the Italian version of the 25-bibitem hikikomori questionnaire. Int J Environ Res Public Health. (2022) 19:13552. 10.3390/ijerph192013552 36294128 PMC9603413

[31] Fino E Iliceto P Carcione A Giovani E Candilera G. Validation of the Italian version of the 25-bibitem hikikomori questionnaire (HQ-25-I). J Clin Psychol. (2023) 79:210–27. 10.1002/jclp.23404 35708975 PMC10083961

[32] Van Krugten FCW Busschbach JJV Versteegh MM Hakkaart-Van Roijen L Brouwer WBF. The mental health quality of life questionnaire (MHQoL): development and first psychometric evaluation of a new measure to assess quality of life in people with mental health problems. Qual Life Res. (2022) 31:633–43. 10.1007/s11136-021-02935-w 34241821 PMC8847188

[33] Enzing JJ Van Krugten FCW Sabat I. Psychometric evaluation of the Mental Health Quality of Life (MHQoL) instrument in seven European countries. Health Qual Life Outcomes. (2022) 20:129. 10.1186/s12955-022-02041-6 36050766 PMC9434504

[34] Stichting Informatie Voorziening Zorg (IVZ). Tussenrapportage Kerncijfers Verslavingszorg 2016-2021. Landelijk Alcohol en Drugs Informatie Systeem. Houten: Stichting IVZ (2023).

[35] Ingram I Kelly PJ Deane FP. Loneliness among people with substance use problems: a narrative systematic review. Drug Alcohol Rev. (2020) 39:447–83. 10.1111/dar.13064 32314504

[36] Rokach A Orzeck T. Coping with loneliness and drug use in young adults. Soc Indic Res. (2003) 61:259–83. 10.1023/A:1021977731756

[37] Søgaard TF Kolind T Haller MB Hunt G. Ring and bring drug services: delivery dealing and the social life of a drug phone. Int J Drug Policy. (2019) 69:8–15. 10.1016/j.drugpo.2019.02.003 31005746

[38] Provost SE Griffin ML Hilton BT. Correlates of optimism among patients in substance disorder inpatient treatment. Am J Addict. (2022) 31:494–501. 10.1111/ajad.13324 35975406

[39] Baumeister RF Leary MR. The need to belong: desire for interpersonal attachments as a fundamental human motivation. Psychol Bull. (1995) 117:497–529. 10.1037/0033-2909.117.3.497 7777651

[40] Allen KA Gray DL Baumeister RF Leary MR. The need to belong: a deep dive into the origins, implications, and future of a foundational construct. Educ Psychol Rev. (2022) 34:1133–56. 10.1007/s10648-021-09633-6 34483627 PMC8405711

[41] Strickland JC Smith MA. The effects of social contact on drug use: Behavioral mechanisms controlling drug intake. Exp Clin Psychopharmacol. (2014) 22:23–34. 10.1037/a0034669 24188170 PMC3926100

[42] Hajema KJ Knibbe RA Drop MJ. Social resources and alcohol-related losses as predictors of help seeking among male problem drinkers. J Stud Alcohol. (1999) 60:120–9. 10.15288/jsa.1999.60.120 10096317

[43] Patrick ME Wightman P Schoeni RF Schulenberg JE. Socioeconomic status and substance use among young adults: a comparison across constructs and drugs. J Stud Alcohol Drugs. (2012) 73:772–82. 10.15288/jsad.2012.73.772 22846241 PMC3410945

[44] Buckner JD Heimberg RG Ecker AH Vinci C. A biopsychosocial model of social anxiety and substance use. Depress Anxiety. (2013) 30:276–84. 10.1002/da.22032 23239365

[45] Zulauf CA Sprich SE Safren SA Wilens TE. The complicated relationship between attention deficit/hyperactivity disorder and substance use disorders. Curr Psychiatry Rep. (2014) 16:436. 10.1007/s11920-013-0436-6 24526271 PMC4414493

[46] Del Boca FK Noll JA. Truth or consequences: the validity of self-report data in health services research on addictions. Addiction. (2000) 95:S347–60. 10.1080/09652140020004278 11132362

[47] Groh DR Ferrari JR Jason LA. Self-reports of substance abusers: the impact of social desirability on social network variables. J Groups Addict Recov. (2009) 4:51–61. 10.1080/15560350802712397 20721306 PMC2922763

[48] Ingram I Kelly PJ Haslam C. Reducing loneliness among people with substance use disorders: feasibility of ‘groups for belonging'. Drug Alcohol Rev. (2020) 39:495–504. 10.1111/dar.13121 32657494

[49] Kim HS Hodgins DC. Component model of addiction treatment: a pragmatic transdiagnostic treatment model of behavioral and substance addictions. Front Psychiatry. (2018) 9:406. 10.3389/fpsyt.2018.00406 30233427 PMC6127248

